# Fluorescence Behavioral Imaging (FBI) Tracks Identity in Heterogeneous Groups of *Drosophila*

**DOI:** 10.1371/journal.pone.0048381

**Published:** 2012-11-07

**Authors:** Pavan Ramdya, Thomas Schaffter, Dario Floreano, Richard Benton

**Affiliations:** 1 Center for Integrative Genomics, Faculty of Biology and Medicine, University of Lausanne, Lausanne, Switzerland; 2 Laboratory of Intelligent Systems, Institute of Microengineering, École Polytechnique Fédérale de Lausanne, Lausanne, Switzerland; Center for Genomic Regulation, Spain

## Abstract

Distinguishing subpopulations in group behavioral experiments can reveal the impact of differences in genetic, pharmacological and life-histories on social interactions and decision-making. Here we describe Fluorescence Behavioral Imaging (FBI), a toolkit that uses transgenic fluorescence to discriminate subpopulations, imaging hardware that simultaneously records behavior and fluorescence expression, and open-source software for automated, high-accuracy determination of genetic identity. Using FBI, we measure courtship partner choice in genetically mixed groups of *Drosophila*.

## Introduction

Natural behavior has evolved in the context of social interactions between conspecifics as well as between species. This is most apparent in the courtship rituals and aggression behaviors observed across the animal kingdom, including in the fruitfly, *Drosophila melanogaster* [1]. Interactions within groups of individuals must therefore be taken into account for a complete understanding of how behavior unfolds. *Drosophila* is poised to reveal important insights in the study of group behaviors as substantial progress in the precision of behavioral quantification has recently been made: Ctrax [2], Cadabra [3], and other software packages enable the semi-automated tracking and analysis of groups and pairs of fruitflies [4]. These tools dramatically expand the potential resolution and sophistication of behavioral studies. However, tracking methods relying on morphological criteria have so far only been able to discriminate large differences between animals, for example smaller males from females [2]. Moreover, morphology is an ambiguous metric because of size variability between strains due to genetic background or culture conditions. Identifying differences using other criteria would bridge a wide methodological gap in *Drosophila*, an organism whose strength lies in the ease of genetic manipulations, by revealing social behaviors and decision-making within groups consisting of individuals of different genotypes and life histories.

**Figure 1:**
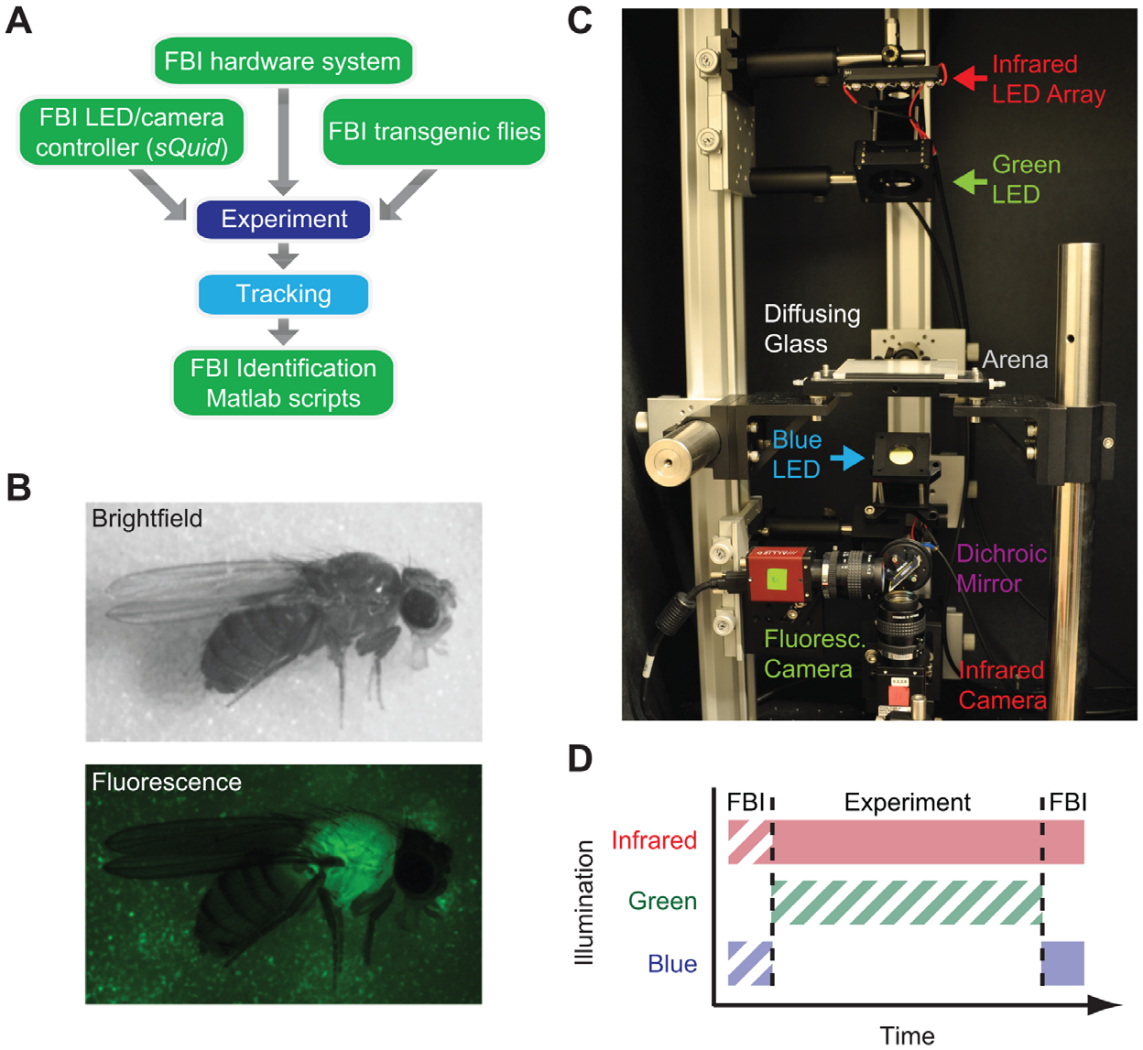
Workflow and tools for Fluorescence Behavioral Imaging (FBI). A) Workflow of FBI experiments. Experiments are performed using an FBI hardware system, *sQuid* software for multi-camera acquisition/LED control, and Actin88F:eGFP transgenic *Drosophila melanogaster*. Subsequently, infrared movies are processed using Ctrax [2] tracking software. FBI post-processing scripts then employ tracking data and fluorescence images to determine the genetic identity of behaving flies in an automated fashion. **B)** Bright-field (top) and fluorescence (bottom) images of an Actin88F:eGFP female fly. **C)** FBI hardware system used in this paper. **D)** Illumination during a single FBI experiment. FBI requires infrared backlight and blue fluorescence illumination following each experiment (solid blocks). Infrared and blue fluorescence illumination prior to each experiment and/or green illumination for vision-dependent behaviors are optional (hatched blocks).

Here we describe Fluorescence Behavioral Imaging (FBI), a toolkit that complements tracking methods by enabling the discrimination of subpopulations within heterogeneous groups of freely behaving flies. FBI bookends behavioral experiments (Figure 1A), making it independent of advances in position/orientation tracking. To discriminate individuals, FBI exploits the expression of a fluorescent protein in a subpopulation, drawing inspiration from physical tagging approaches that are used in larger insects [5] and leveraging the power of *Drosophila* genetics. By analogy with clonal cellular analyses using fluorescent markers in *Drosophila* [6], FBI also confers the advantages inherent in allowing the phenotypic comparison of two distinct populations of animals within the same experiment. Although this approach is imminently scalable to discriminate many subpopulations using multiple fluorophores, here we illustrate the distinction of two subgroups of flies using a single fluorophore, enhanced Green Fluorescent Protein (eGFP).

## Results and Discussion

### Tools for Fluorescence Behavioral Imaging (FBI)

To tag one subgroup of flies we generated transgenic animals expressing eGFP under the control of the *actin88F* promoter, which drives expression in indirect flight muscles of the thorax [7] (Figure 1B). This approach confers an advantage over a ubiquitous expression strategy since ubiquitous expression of eGFP using a Tubulin promoter sequence can result in changes in basal locomotion (data not shown) while the translational velocity, angular velocity, and courtship duration of Actin88F:eGFP flies and control flies are indistinguishable (Figure S1). Homozygous Actin88F:eGFP flies are fecund and able to fly but assay specific controls should not be neglected in new experimental scenarios requiring high precision behavioral measurements. Another advantage of this transgene expression pattern is that the spatial intensity distribution of thoracic eGFP fluorescence is distinct from typical cuticular auto-fluorescence, facilitating discrimination of eGFP-expressing flies (GFP) from those lacking this transgene (non-GFP) flies.

**Figure 2:**
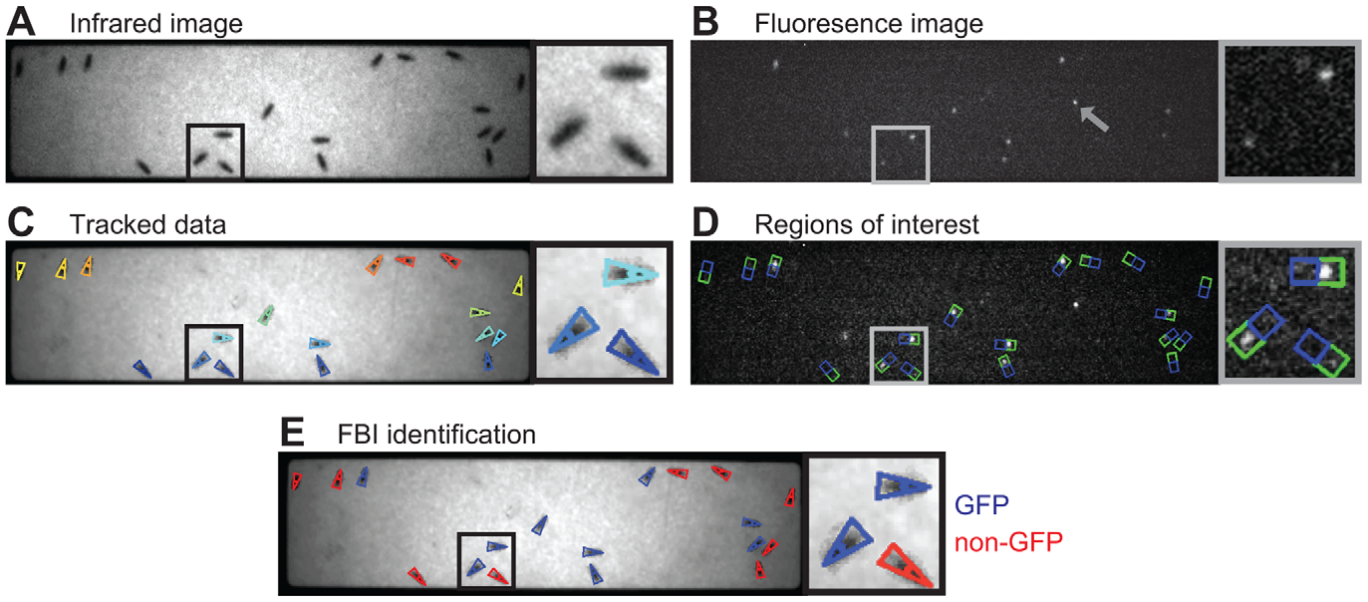
Processing of video images to yield genetic identities in heterogeneous groups of *Drosophila*. A) Infrared backlit image of a genetically heterogeneous group of flies. Black box indicates the inset to the right of the image. **B)** Fluorescence image of the same group of flies. Grey box indicates inset. Note that extraneous autofluorescence from the experimental arena (grey arrowhead) does not impede subsequent analysis. **C)** Infrared image overlaid with size, position and orientation tracking data acquired using Ctrax [2]. Each colored triangle corresponds to a single fly. **D)** Fluorescence image overlaid with Regions of interest (ROIs). Green boxes encompass Front ROIs and blue boxes Rear ROIs for each fly. **E)** Infrared image overlaid with FBI data identifying each fly as GFP (blue) or non-GFP (red).

To simultaneously access genetic and positional information, we developed a macroscopic imaging system for synchronous fluorescence and infrared (IR) backlight video recording (Figure 1C). Due to the effect of visible light on locomotion [8], it is less intrusive to perform fluorescence imaging only after the completion of each experiment (Figure 1D). However, to explore the robustness of our method under multiple conditions, we synchronously recorded images from IR backlight illumination (Figure 2A) and visible eGFP excitation (Figure 2B) for a brief period (10 seconds) both prior to and following each experiment. To coordinate the timing of LED activation and camera acquisition we developed open-source software called *sQuid* (available at: http://lis.epfl.ch/squid), which permits the control of multiple cameras and a computer output interface with millisecond temporal precision. Using these tools, we recorded groups of eighteen freely walking flies in an enclosed arena for up to five minutes. Following each experiment IR videos were tracked using Ctrax [2]. Using tracking data (Figure 2C), we could delineate regions of interest (ROI) for each fly in each fluorescence image (Figure 2D). For subsequent analysis this region (Total ROI) was divided into a subregion containing the head and thorax (Front ROI; Figure 2D, green) and a second subregion containing the abdomen (Rear ROI; Figure 2D, blue).

### Automation of Genotypic Identification

**Figure 3:**
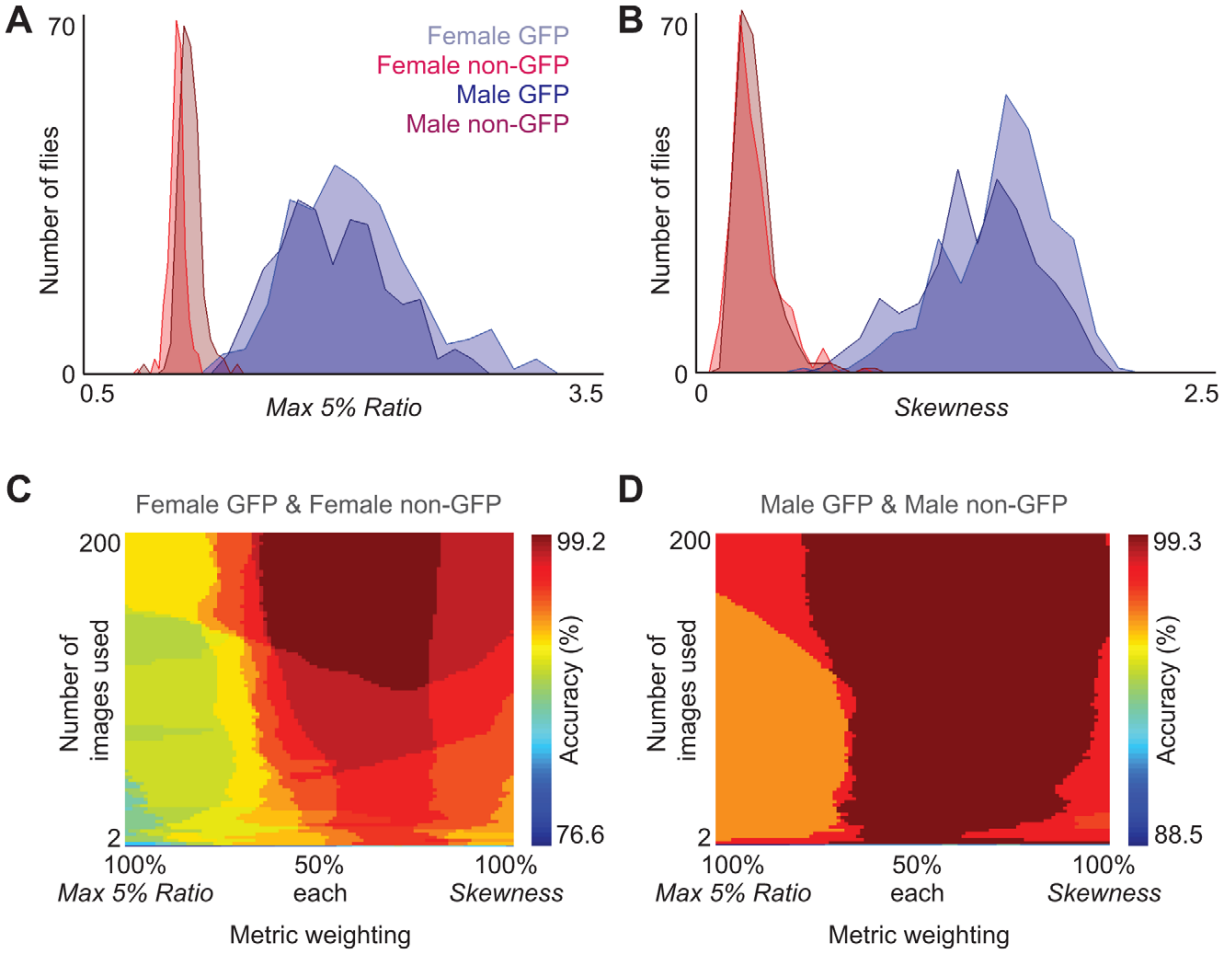
Best fluorescence discrimination metrics and their accuracy landscapes when used in combination. **A)** Histograms of the *Max 5% Ratio* metric for GFP females (light blue, n = 270 flies), non-GFP females (red, n = 267 flies), GFP males (dark blue, n = 270 flies), non-GFP males (dark red, n = 268 flies). Here the mean of the maximum 5% pixel values in the Front ROI is divided by the mean of the maximum 5% pixel values in the Rear ROI. Data is taken from homogeneous group experiments. **B)** Histograms of the *Skewness* metric for the same dataset. Here the skewness of pixel value distributions in each Total ROI is measured. **C)** Fluorescence discrimination accuracy of heterogeneous groups of female flies (n = 14 experiments; GFP females, n = 123; non-GFP females, n = 125) or **D)** male flies (n = 15 experiments; GFP males, n = 142; non-GFP males, n = 136). Here the number of flies expected in each genotype is incorporated into the genotype discrimination algorithm. X-axes show the weighting of each metric. Y-axes show the cumulative number of images averaged for metric measurements. Color bars indicate the discrimination accuracy.

While in principle these images can be used to discriminate between GFP and non-GFP subpopulations by eye (Figure 2E; GFP in blue, non-GFP in red), such an approach is very time consuming and susceptible to human error. We therefore developed FBI post-processing Matlab scripts for automatically discriminating genetic identity with high accuracy (see Text S1 for details; scripts available at http://lis.epfl.ch/FBI). We began by measuring the range of fluorescence values for GFP or non-GFP flies. After recording IR and fluorescence videos of genetically homogeneous groups, pixel values were extracted from Front and Rear ROIs for each fly in fluorescence images. Next we evaluated fifteen quantitative metrics for their accuracy (Figure S2) in processing fluorescence pixel values to produce a result that is above a threshold (Figure S3) for GFP flies and below this threshold for non-GFP flies. We identified two metrics that most effectively separated pixel value histograms for GFP and non-GFP flies into non-overlapping distributions (Figure 3A, B). The first metric, *Max 5% Ratio*, is the mean of the brightest 5% of pixel values in the Front ROI divided by the mean of the brightest 5% of pixel values in the Rear ROI (Figure 3A). This ratiometric normalization reduces the impact of variability in GFP excitation and expression levels. The second metric, *Skewness*, is a statistical measure of the pixel value distribution for the Total ROI (Figure 3B, see Materials and Methods for mathematical formulation). These two metrics are dimensionless making them more robust to hardware and illumination differences across experimental platforms.

We discovered that these two metrics were also complementary: each provided optimal discrimination for different mixtures of fly genders and fluorescence expression (Figure S2). Such complementarity suggested that these metrics might be even more effective when used in combination. By systematically testing different proportions of the two metrics with different discrimination thresholds on all genotypic mixture combinations, we confirmed that higher and more robust discrimination accuracy could be achieved with a combination of both metrics rather than one alone (Figures S4 & S5).

To test this automated approach for discriminating genetic identity in heterogeneous groups of flies, we performed experiments using GFP and non-GFP females or males together (female-female: n = 123 GFP flies and 125 non-GFP flies from 14 experiments; male-male: n = 142 GFP flies and 136 non-GFP flies from 15 experiments). Using optimal proportions and discrimination thresholds derived from homogeneous group experiments (Figure S4), we could accurately identify GFP expression in heterogeneous groups of flies. To achieve >90% discrimination accuracy in both experiments, only 4 images were needed, while 20 images brought accuracy to above 95% (Figure S6 insets). However, achieving >99% discrimination accuracy required 602 images for female flies (Figure S6A, more than males due to abdominal autofluorescence) and 386 images in male flies (Figure S6B). Such high performance might therefore require prohibitively long eGFP excitation periods for light-sensitive experiments at our frame-rate of 20 frames per second.

To overcome this problem, we reasoned that incorporating prior information of the expected number of GFP and non-GFP flies might reduce the number of images needed for high accuracy discrimination. We used this additional information by sorting processed fluorescence values for each fly in descending order and then dividing this list in two. The top portion denoted putative GFP flies (based on the expected number) while the lower denoted putative non-GFP flies. By exploring the dependence of discrimination accuracy on the weighting of each metric and the number of images used, we observed that this strategy could reach >99% discrimination accuracy with fewer images (102 images in females and 4 images in males) and using a wide range of metric weightings (Figure 3C, D). Importantly, >99% discrimination accuracy could also be achieved with FBI only after each experiment (134 images in females and 2 images in males, Figure S7) precluding the requirement for blue light illumination prior to experimental recordings, which could potentially influence locomotor and other behaviors [8]. In summary, using a combination of complementary pixel value metrics as well as prior knowledge of the proportion of labelled flies, FBI post-processing scripts can achieve high accuracy automated identification requiring only a brief period of fluorescence imaging at the beginning and/or end of each experiment.

### Measuring Courtship Choice Using FBI

**Figure 4:**
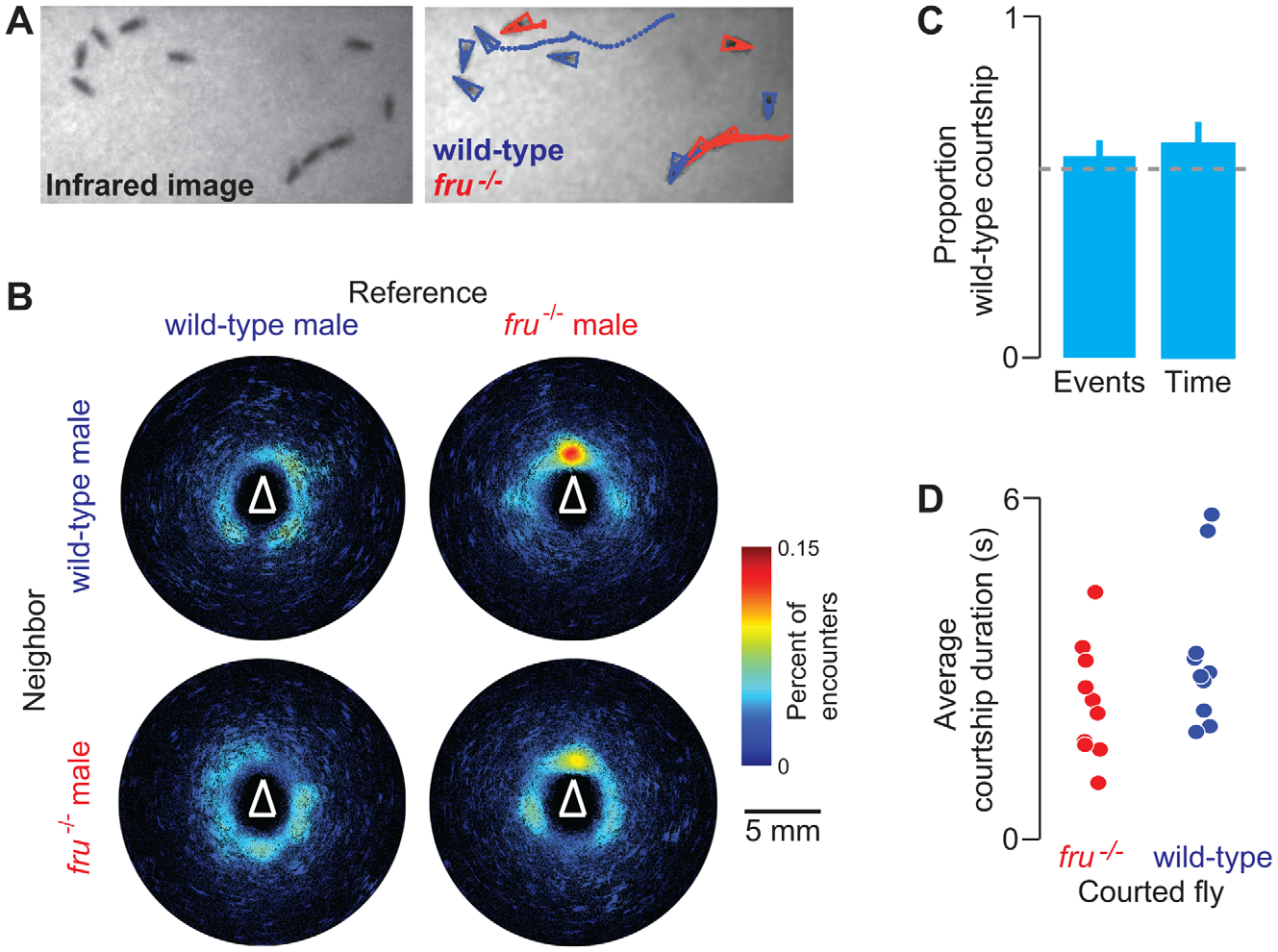
FBI analysis of courtship interactions among mixed groups of *fruitless* (*fru^−/−^*) and wild-type males. A) Raw (left) and Ctrax/FBI (right) images of courtship interactions between wild-type GFP males (blue) and *fru^−/−^* non-GFP males (red) (flies are trailed by colored dots indicating their position in the previous 50 images/2.5 s). Three flies at the bottom-right form a courtship chain, as *fru^−/−^* mutants court other males. **B)** Encounter density heat maps for wild-type reference flies with respect to other wild-type neighbors (top-left), *fru^−/−^* reference flies with respect to wild-type neighbors (top-right), wild-type reference flies with respect to *fru^−/−^* neighbors (bottom-left), and *fru^−/−^* reference flies with respect to *fru^−/−^* neighbors (bottom-right); n = 10 experiments; 60 *fru^−/−^* flies and 60 GFP flies. Color bar indicates the percent of encounters observed at a given pixel. White triangles denote the orientation and approximate size of reference flies. **C)** Bar plots indicating the proportion of *fru^−/−^* courtship events and courtship time towards wild-type flies (n = 10 experiments, mean and s.e.m.; chance based on the proportion of wild-type flies indicated in grey dashed line). **D)** The average duration of each courtship event of *fru^−/−^* flies (n = 10 experiments) towards *fru^−/−^* males (red) or wild-type males (blue).

Tracking algorithms allow high-throughput quantitative measurements of behavior but cannot resolve differences in genotype or life-history. Consequently, large-scale studies requiring mixed populations such as those measuring social decision-making are out of reach. To illustrate how FBI overcomes this limitation, we studied courtship choice in genetically heterogeneous groups of male flies. We examined the initial chasing/orienting steps of the courtship ritual in males mutant for *fruitless* (*fru^−/−^)*, which lack an important genetic determinant of sexual behavior [9]. *fru^−/−^* males have altered sexual orientation, and court other males. It is not known whether *fru^−/−^* mutants prefer to court wild-type males (which normally rebuff homosexual advances) or other *fru^−/−^* mutants, which might be more receptive to courtship. We therefore tested whether *fru^−/−^* males preferred to court wild-type males over other *fru^−/−^* males when mixed in groups of twelve (n = 10 experiments). We confirmed that *fru^−/−^* males court other males, sometimes forming “chains” that incorporate both wild-type and *fru^−/−^* mutant animals (Figure 4A). This can also be visualized in encounter density plots [2] showing a dramatically high proportion of *fru^−/−^* male encounters occurring near the head since sensory cues promoting courtship are detected by neurons on the head or forelegs (Figure 4B, right). When we quantified the proportion of courtship events (Figure 4C, left) and courtship time (Figure 4C, right) as well as courtship event duration (Figure 4D), we observed that *fru^−/−^* males courted wild-type and *fru^−/−^* flies with similar intensity (Student’s t-test, P = 0.37 for events and P = 0.23 for time compared to chance; Wilcoxon rank-sum test, P = 0.14 for event duration). These data suggest that at least the initial courtship decisions of *fru^−/−^* males are not strongly influenced by partner behavior and that receptivity of males to initial advances by other males is not altered in *fru^−/−^* mutants.

## Discussion

FBI can be used to complement tracking methods, providing a general way to link quantitative *Drosophila* group behavior with subgroup specific experimental perturbations such as genetic mutations or life-history modifications such as drug treatments. We envision that this approach could be easily applied to the behavioral analysis of any species amenable to transgenesis and tracking (e.g. mosquitoes [10], *C. elegans* [11], zebrafish [12], and mice [13]). Additionally, it could be modified to incorporate a wealth of fluorescent tools towards the study of behavior. For example, one might tag more than two subgroups using multiple fluorophores [14], measure gene expression during behavior [15], use fluorophore photo-activation [16] for behavior-triggered marking, or study real-time feeding by measuring the ingestion of synthetic fluorescent dyes.

## Materials and Methods

### Molecular Biology

For the Actin88F:eGFP construct, a 2053 bp region immediately upstream of the *actin88F* gene was amplified by the Expand High Fidelity PLUS PCR system (Roche) from Oregon-R genomic DNA using the following forward primer containing a *BmtI* site: 5′-GCT AGC ATG CAC AAT AGG CAA ATT TAG TT-3′ and reverse primer containing an *EcoRI* site: 5′-GAA TTC CTT GGC AGT TGT TTA TCT GGA A-3′. eGFP was similarly amplified using the following forward primer containing a *KpnI* site: 5′-GGT ACC ATG GTG AGC AAG GGC GA-3′ and reverse primer containing an *XbaI* site: 5′-TCT AGA TTA CTT GTA CAG CTC GTC CAT GC-3′. PCR products were T:A cloned into pGEM-T Easy (Promega), sequenced, and subcloned with *BmtI* and *EcoRI* into the pattB vector [17]. *eGFP* was subsequently amplified and subcloned downstream of this promoter fragment.

### Drosophila Strains

Transgenic Actin88F:eGFP strains (“GFP” flies) were generated (Genetic Services, Inc., Cambridge, Massachusetts, USA) with the phiC31-based integration system using attP40 (second chromosome) or attP2 (third chromosome) landing sites [18]. To examine a worst-case-scenario for discrimination analysis, potentially encountered in the context of experiments with flies carrying other transgenes, in most experiments we used “non-GFP” flies with a Minos transposable element insertion in the *IR64a* locus (*IR64a^mi^*) [19] whose marker drives GFP expression in the ocelli and eyes. *fruitless* mutant flies (*fru^−/−^*) were homozygous *fru* ^Gal4^ [20]. All flies were back-crossed to *w^1118^* for five generations and self-crossed to achieve homozygosity. For courtship control experiments, GFP males were compared to *w^1118^* males.

### Fluorescence Behavioral Imaging (FBI) System

The experimental arena consisted of an 80 mm×20 mm enclosure with a height of 1.3 mm restricting flies to walking in two-dimensions (custom designed and machined from polyoxymethylene and acrylic glass). To achieve spectral separation of the two channels for each camera (Allied Vision Technologies, Stadtroda, Germany), we used a 580 nm long-pass dichroic filter (F38-580 HC beamsplitter BS 580, AHF analysentechnik, Germany) to pass infrared (IR) photons emitted from back-light illuminating 850 nm IR LEDs (IR-1WS-850-w/Star, Super Bright LEDs Inc. St. Louis Missouri, USA) through a diffusing glass (ThorLabs, USA) to a camera bearing a 785 nm IR long-pass filter (F76-787 Edge Basic Long Pass, AHF analysentechnik, Germany). This dichroic also reflected photons below 580 nm into a camera bearing a GFP band-pass filter (AHF analysentechnik, Germany). GFP was excited using a panel of blue super-bright 470 nm LEDs (LED470-66-60, Roithner Lasertechnik GmbH, Germany) placed incident to the behavioral arena.

### Behavioral Experiments

All experiments were performed on 2 day post-eclosion adult *Drosophila* raised at 25°C on a 12 h light:12 h dark cycle. Experiments were performed in a temperature-controlled room at 25°C.

#### Homogeneous group FBI experiments

These experiments (Figures 3A, B; S1A, B; S2, S3, S4, S5) used 18 flies (either all male or all female; either all GFP or all non-GFP) and were performed as follows: GFP/IR imaging (10 s) – IR imaging (1 min) – GFP/IR imaging (10 s).

#### Heterogeneous group FBI experiments

These experiments (Figures 3C, D; S6, S7) used 18 flies (either all male or all female; half GFP and half non-GFP) and were performed as follows: GFP/IR imaging (1 min).

#### fru^−/−^/wild-type group FBI experiments

These experiments (Figure 4) used 12 flies (all male; half *fru^−/−^* and half GFP wild-type) and were performed as follows: GFP/IR imaging (10 s) – IR imaging (5 min) – GFP/IR imaging (10 s).

#### Courtship control experiments

These experiments (Figure S1C) used 2 flies (1 intact male and 1 headless female as in [21]) and were performed as follows: IR imaging (20 min). Male courtship behavior (defined as proximity/licking, wing-extension, or mounting) was manually scored.

Following all FBI experiments, Ctrax [2] was run on IR video data to obtain the position, orientation, and size of each fly. These data were then used to construct rectangular regions of interest (ROIs) on GFP fly images for subsequent analyses using custom-written shell scripts and Matlab scripts (The Mathworks, Natick, Massachusetts, USA). These scripts are freely available at freely available at http://lis.epfl.ch/FBI.

### Automation Metric Evaluation Using Homogeneous Group Data

Homogeneous groups of flies were used for metric evaluations (Figure S2, S3, S4, S5) to ensure genotype identity and to provide a model for the distribution of data values. After tracking, a vector of pixel values from Total, Front, and Rear ROIs were extracted for each fly in each image (Figure 2D). Metrics were used to process these pixel values. One-thousand threshold values within the possible range were tested on the output of each metric. Flies with metric values above a given threshold were assigned the identity of *GFP fly* while those below this threshold were assigned the identity of *non-GFP fly*. These assignments were tested against the known genotype of each fly to determine the error or, inversely, the discrimination accuracy (100% - error%). Our comprehensive evaluations yielded two metrics with best discrimination accuracy: *Max 5% Ratio* and *Skewness* (Figure S2). *Max 5% Ratio* is the time-averaged mean of the maximum 5% pixel values in the Front ROI divided by the time-averaged mean of the maximum 5% pixel values in the Rear ROI. The ratio of single maximum pixel values (*Maximum Front ROI/Maximum Rear ROI*) performed equally well but was not selected due to low robustness against pixel value noise. *Skewness* was measured using the Matlab function of the same name and is defined as follows:
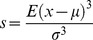
Where the skewness *s*, is defined by the mean of the data *x*, *μ*, the standard deviation of *x*, *σ*, and the expected value of *t*, *E(t)* [22].

### Automation Tests Using Heterogeneous Group Data

For heterogeneous group experiments, the fluorescence identity ground-truth for each fly was obtained by human observer evaluation of videos with ROIs superimposed on GFP images. When the algorithm did not take the known number of GFP flies into account (Figure S6), it used proportions and thresholds that generated the largest cross-section of maximum accuracy regions in the discrimination accuracy heat maps from homogeneous group experiments (Figure S5). When the algorithm took the known number of GFP flies into account (Figure 3C, D & Figure S7), for each experiment, values for each fly obtained using the mixture of metrics were sorted in descending order. The top N flies, where N is the number of GFP flies expected, were assigned the identity *GFP fly*, while those remaining were assigned the identity *non-GFP fly*. Image-number analyses (Figure 3) were performed using data from both the beginning and the end of each experiment to exploit fly movement and reduce the impact of spatial inhomogeneity in fluorescence illumination. For example, when two images were used, one image was taken from the start of the experiment and one was taken from the end. Additional image-number analyses (Figure S7) only took images from the end of the experiment. All measurements and evaluations were performed using custom-written Matlab scripts (The Mathworks, Massachusetts, USA).

### FBI Courtship Experiment Analysis

For FBI male-male courtship experiments, videos were first processed using Ctrax and FBI post-processing scripts to derive the behavioral statistics and genotypic identity of each fly. Subsequently, videos with tracking/genotypic identity overlaid (a modification of Ctrax’s *showtrx.m* script named *showtrx_GENO.m* available at: lis.epfl.ch/FBI) were manually annotated for courtship chasing/orientation events. For each event, the genetic identity of the chase target and the duration of the chase were noted. Data were tested for normality using the Lilliefors test. Normally distributed courtship probability and duration data were analyzed using the Student’s t-test. Non-normal chase duration data were compared using the Wilcoxon rank sum test.

## Supporting Information

Figure S1 **Global** **behavior is indistinguishable between GFP and non-GFP flies. A)** Mean velocity and **B)** Mean absolute angular velocity – without respect to direction of turning – per fly and per experiment for each genotype and sex (Wilcoxon rank sum test: Velocity: female GFP v. female non-GFP P = 0.97, male GFP v male non-GFP P = 0.59; Turning: female GFP v. female non-GFP P = 0.51, male GFP v. male non-GFP P = 0.25; female GFP: n = 270 flies from 15 experiments; female non-GFP: n = 267 flies from 15 experiments; male GFP: n = 270 flies from 15 experiments; male non-GFP: n = 268 flies from 15 experiments). **(C)** Male courtship duration for each genotype (Wilcoxon rank sum test: GFP v. non-GFP p = 0.29; n = 77 and 75 respectively).
(TIF)

Figure S2 **Fluorescence discrimination accuracy for each metric.** All tested metrics and their corresponding discrimination errors for four types of experiments: female GFP and female non-GFP, male GFP and male non-GFP, female GFP and male non-GFP, male GFP and female non-GFP. Metrics are grouped into classes based on qualitative similarity. Red values indicate the best performers for a given experiment type (the lowest sum of false positives and false negatives). Gray shading indicates the two metrics used subsequently.
(TIF)

Figure S3 **Thresholds achieving best discrimination accuracy for each metric.** All tested metrics and thresholds corresponding to their best fluorescence discrimination for four types of experiments: female GFP and female non-GFP, male GFP and male non-GFP, female GFP and male non-GFP, male GFP and female non-GFP. Metrics are grouped into classes based on qualitative similarity. Gray shading indicates the two metrics used subsequently.
(TIF)

Figure S4 **Higher discrimination accuracies can be obtained by using a combination of metrics.** Best discrimination using both *Max 5% Ratio* and *Skewness* in varying amounts. Indicated are error rate (red indicates best discrimination accuracy for each experiment type), best weighting for each metric (percent of total), and best thresholds for discrimination using this combination.
(TIF)

Figure S5 **Discrimination accuracy for a combination of metrics as a function of metric weighting and discrimination threshold.** Accuracy of discriminating between histograms of metric values of GFP and non-GFP flies that are, respectively, **A)** female-female, **B)** female-male, **C)** male-female, and **D)** male-male (n = 15 experiments each). X-axes show the weighting of each metric. Y-axes show the cut-off threshold applied to separate GFP from non-GFP data. Color bars show discrimination accuracy range. Black dashed lines indicate the empirical optima (maximum cross-section) for thresholds and metric weighting.
(TIF)

Figure S6 **Discrimination accuracy as a function of the number of images used.** Dependence of discrimination accuracy on the cumulative number of images used for data-averaging in **A)** female-female (n = 14 experiments; GFP females, n = 123; non-GFP females, n = 125) and **B)** male-male heterogeneous group experiments (n = 15 experiments; GFP males, n = 142; non-GFP males, n = 136). Metric weights are taken from homogeneous experiment analyses. Inset is a zoom into the first 20 images (note different y-axes). Red line: percent accurate non-GFP identifications, blue line: percent accurate GFP identifications, dashed black line: percent overall accuracy.
(TIF)

Figure S7 **Discrimination accuracy when restricting FBI to the period after each experiment.** Dependence of discrimination accuracy on metric weighting and the cumulative number of images used for data-averaging in **A)** female-female (n = 14 experiments; GFP females, n = 123; non-GFP females, n = 125) and **B)** male-male (n = 15 experiments; GFP males, n = 142; non-GFP males, n = 136) heterogeneous group experiments. Analyses employ only FBI data taken after each behavioral experiment. The number of flies expected in each genotype is incorporated into the discrimination algorithm. X-axes show the weighting of each metric. Y-axes show the cumulative number of images averaged for metric measurements. Color bars indicate the discrimination accuracy.
(TIF)

Text S1 Acquiring FBI data with *sQuid* and details regarding FBI Matlab m-files for post-processing FBI data.
(PDF)
