# Supplementary figures and images for: Fluorescence Behavioral Imaging (FBI) Tracks Identity in Heterogeneous Groups of Drosophila
Source: PLoS One. 2012 Nov 7;7(11):e48381. doi: 10.1371/journal.pone.0048381 (PMC3492344; doi:10.1371/journal.pone.0048381)

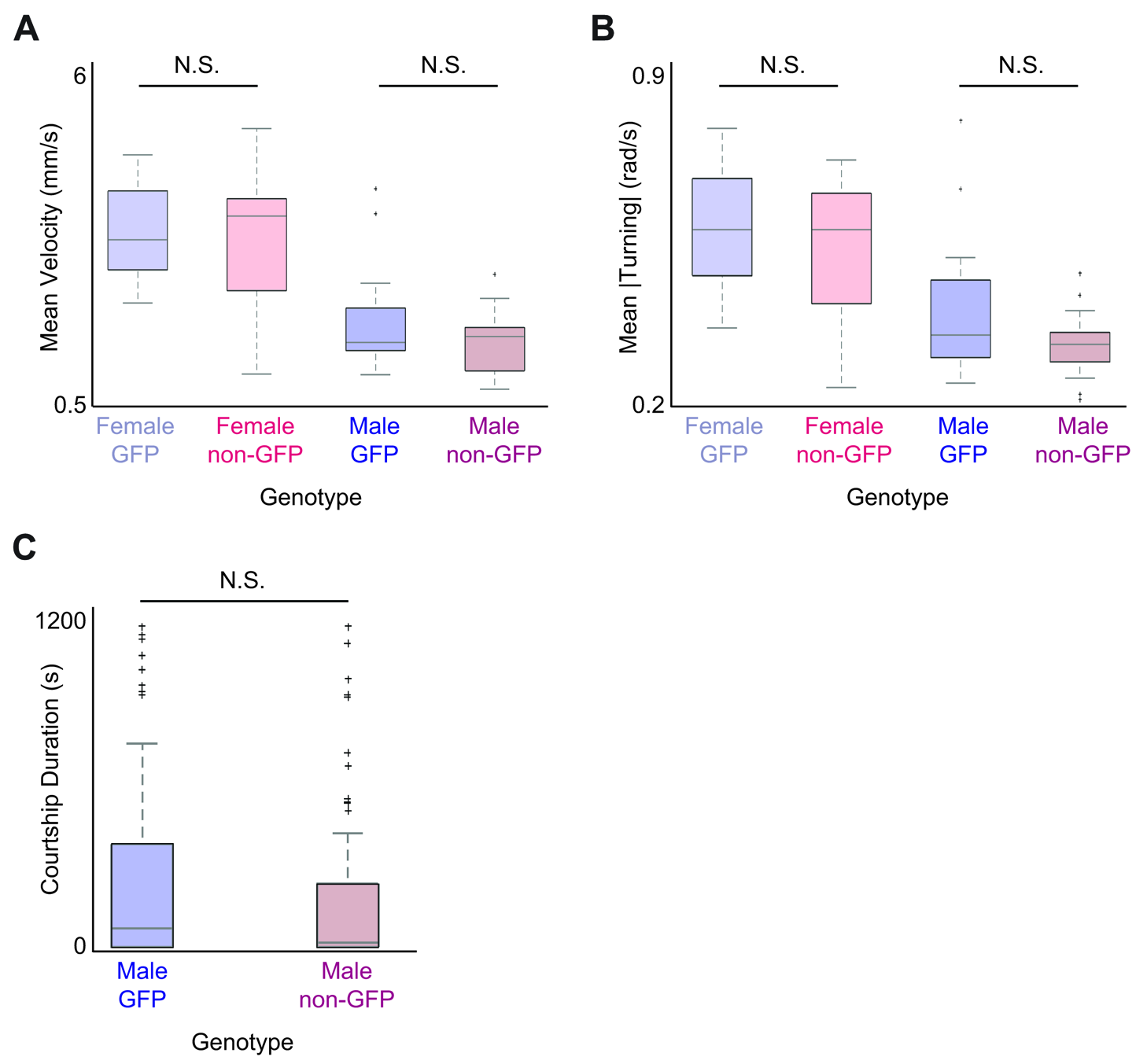

Supplement: Figure S1 — Global behavior is indistinguishable between GFP and non-GFP flies. A) Mean velocity and B) Mean absolute angular velocity – without respect to direction of turning – per fly and per experiment for each genotype and sex (Wilcoxon rank sum test: Velocity: female GFP v. female non-GFP P = 0.97, male GFP v male non-GFP P = 0.59; Turning: female GFP v. female non-GFP P = 0.51, male GFP v. male non-GFP P = 0.25; female GFP: n = 270 flies from 15 experiments; female non-GFP: n = 267 flies from 15 experiments; male GFP: n = 270 flies from 15 experiments; male non-GFP: n = 268 flies from 15 experiments). (C) Male courtship duration for each genotype (Wilcoxon rank sum test: GFP v. non-GFP p = 0.29; n = 77 and 75 respectively). (TIF) [file pone.0048381.s001.tif]

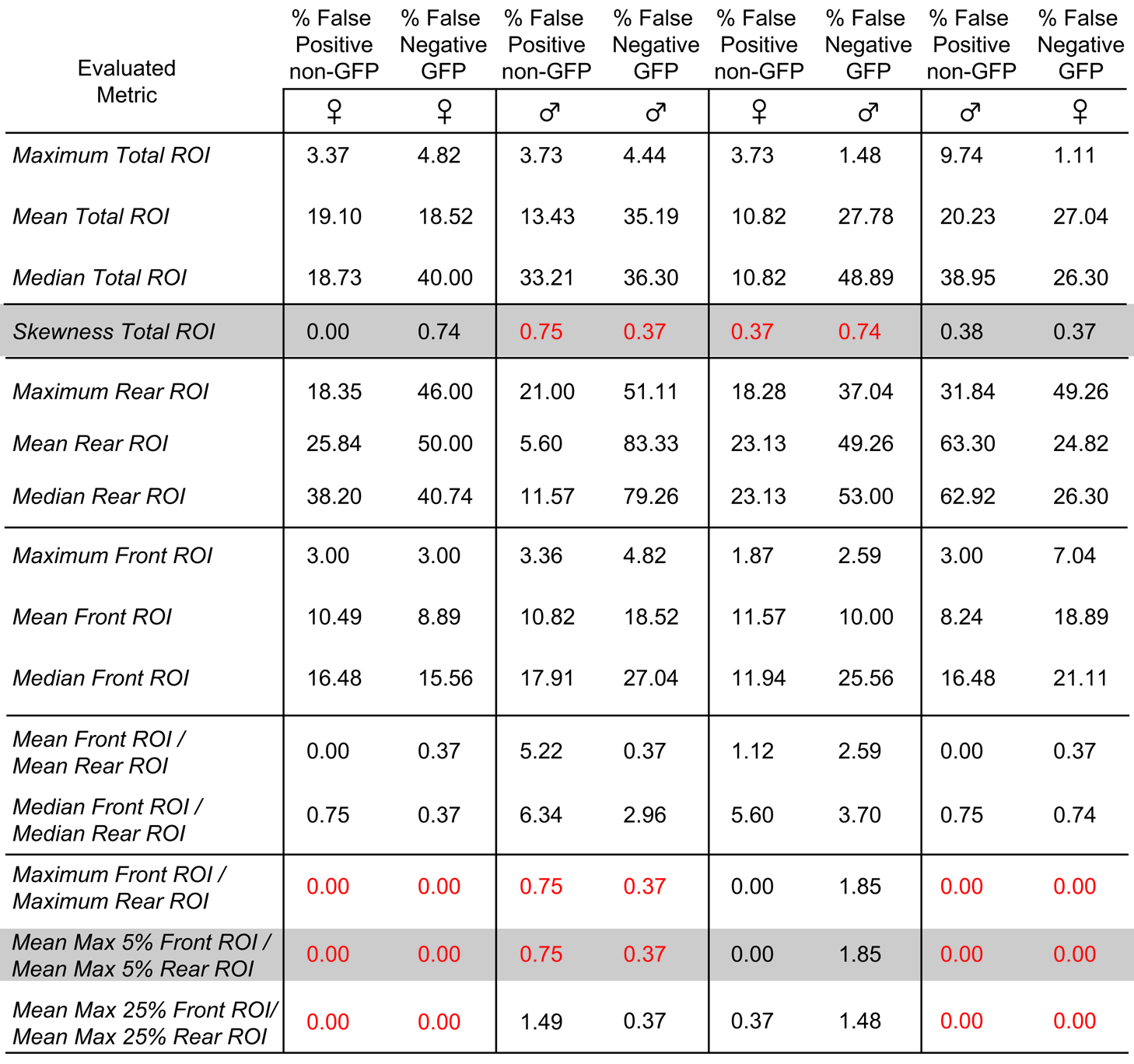

Supplement: Figure S2 — Fluorescence discrimination accuracy for each metric. All tested metrics and their corresponding discrimination errors for four types of experiments: female GFP and female non-GFP, male GFP and male non-GFP, female GFP and male non-GFP, male GFP and female non-GFP. Metrics are grouped into classes based on qualitative similarity. Red values indicate the best performers for a given experiment type (the lowest sum of false positives and false negatives). Gray shading indicates the two metrics used subsequently. (TIF) [file pone.0048381.s002.tif]

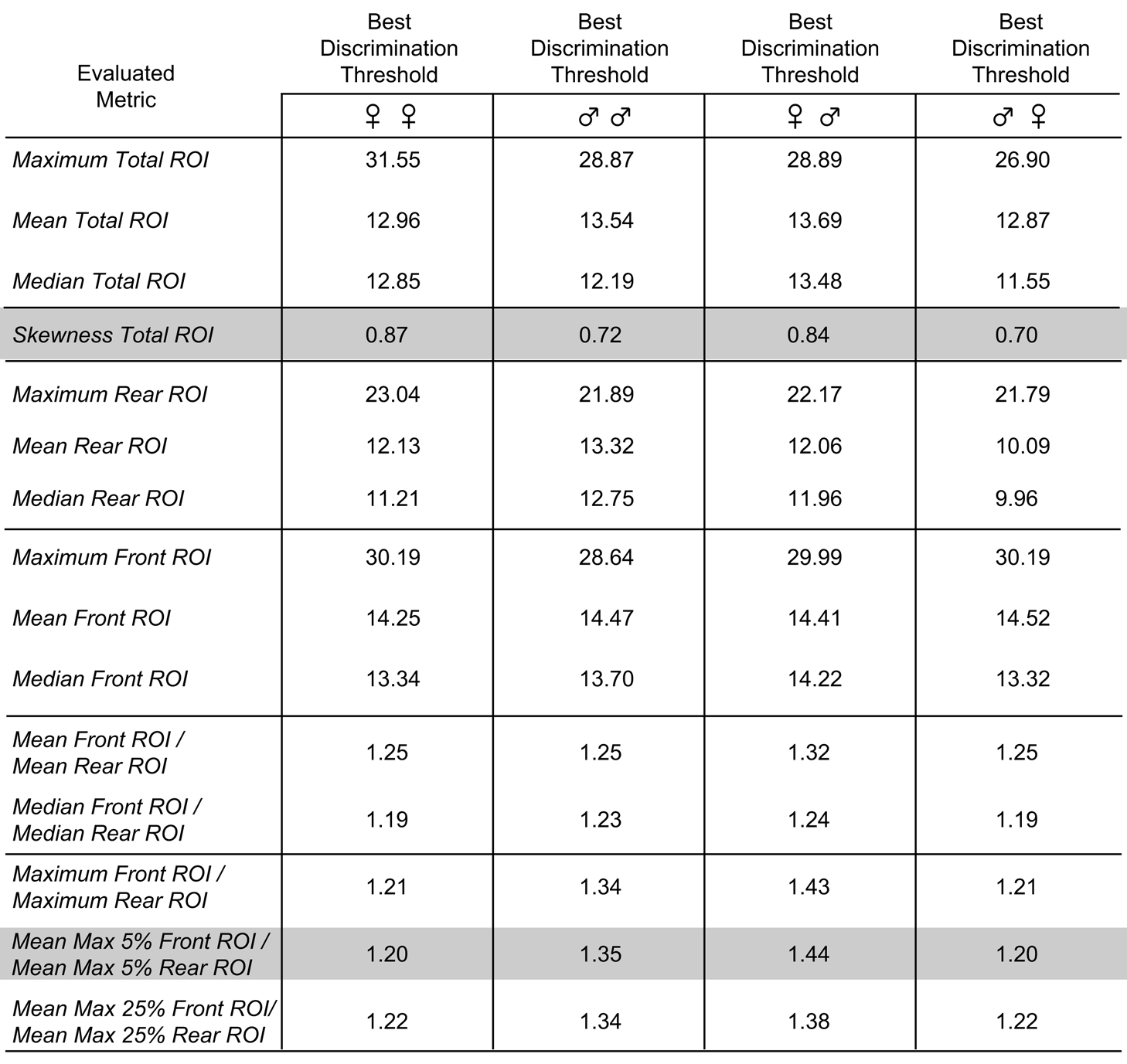

Supplement: Figure S3 — Thresholds achieving best discrimination accuracy for each metric. All tested metrics and thresholds corresponding to their best fluorescence discrimination for four types of experiments: female GFP and female non-GFP, male GFP and male non-GFP, female GFP and male non-GFP, male GFP and female non-GFP. Metrics are grouped into classes based on qualitative similarity. Gray shading indicates the two metrics used subsequently. (TIF) [file pone.0048381.s003.tif]

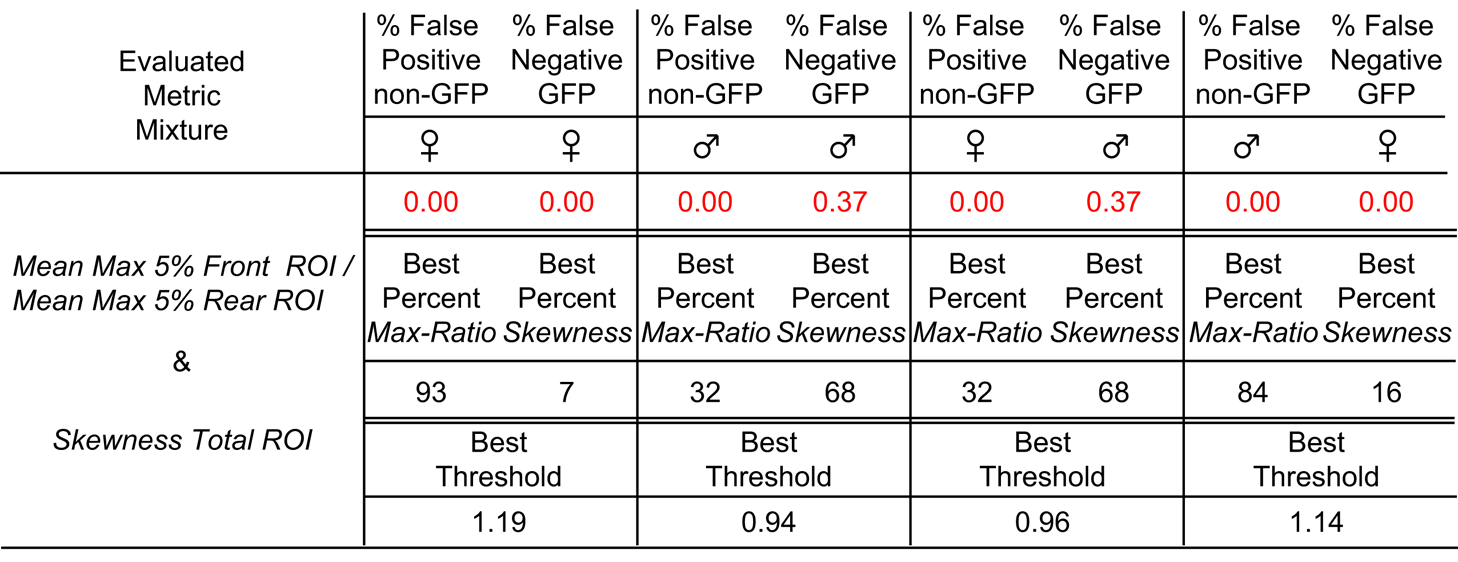

Supplement: Figure S4 — Higher discrimination accuracies can be obtained by using a combination of metrics. Best discrimination using both Max 5% Ratio and Skewness in varying amounts. Indicated are error rate (red indicates best discrimination accuracy for each experiment type), best weighting for each metric (percent of total), and best thresholds for discrimination using this combination. (TIF) [file pone.0048381.s004.tif]

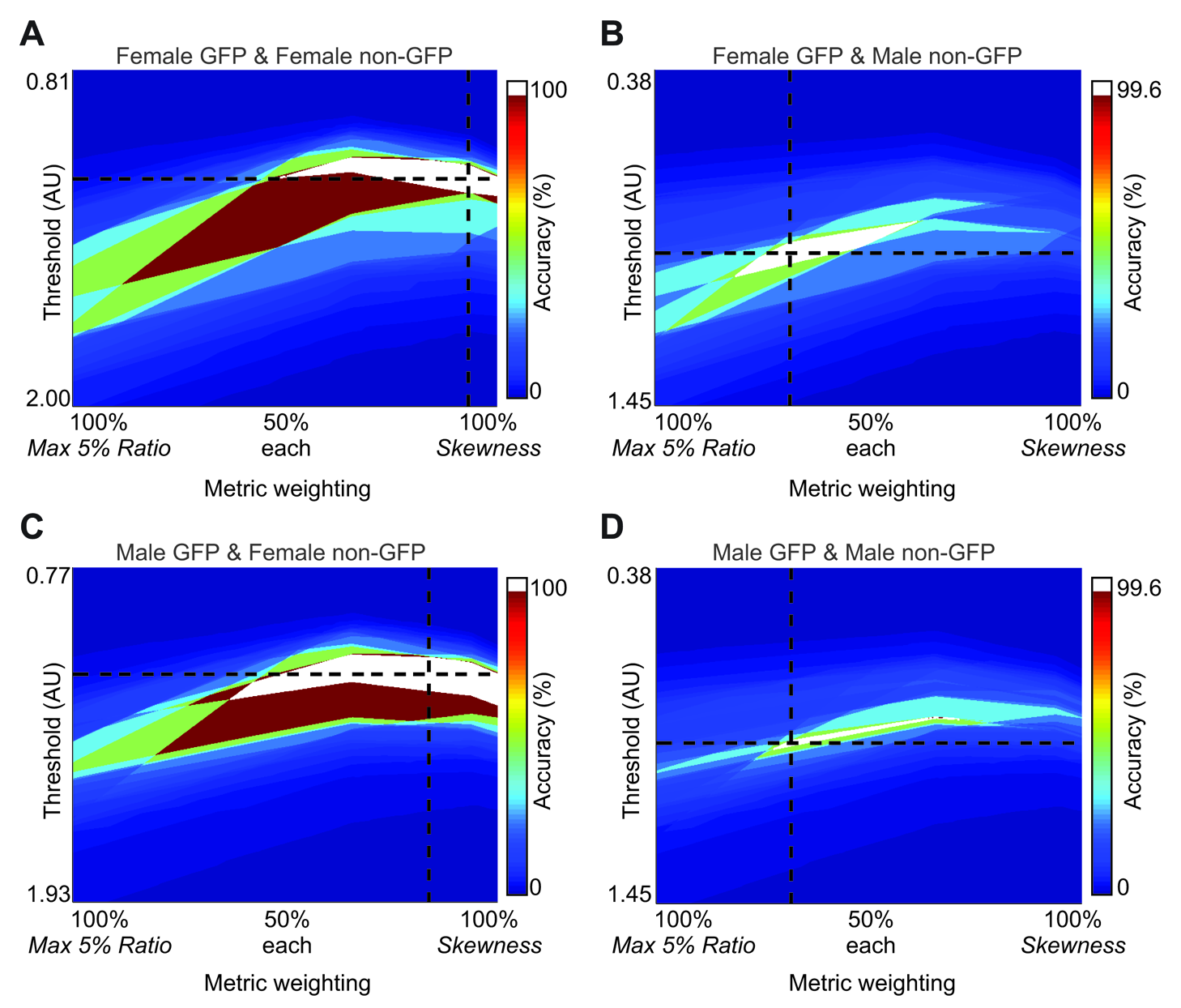

Supplement: Figure S5 — Discrimination accuracy for a combination of metrics as a function of metric weighting and discrimination threshold. Accuracy of discriminating between histograms of metric values of GFP and non-GFP flies that are, respectively, A) female-female, B) female-male, C) male-female, and D) male-male (n = 15 experiments each). X-axes show the weighting of each metric. Y-axes show the cut-off threshold applied to separate GFP from non-GFP data. Color bars show discrimination accuracy range. Black dashed lines indicate the empirical optima (maximum cross-section) for thresholds and metric weighting. (TIF) [file pone.0048381.s005.tif]

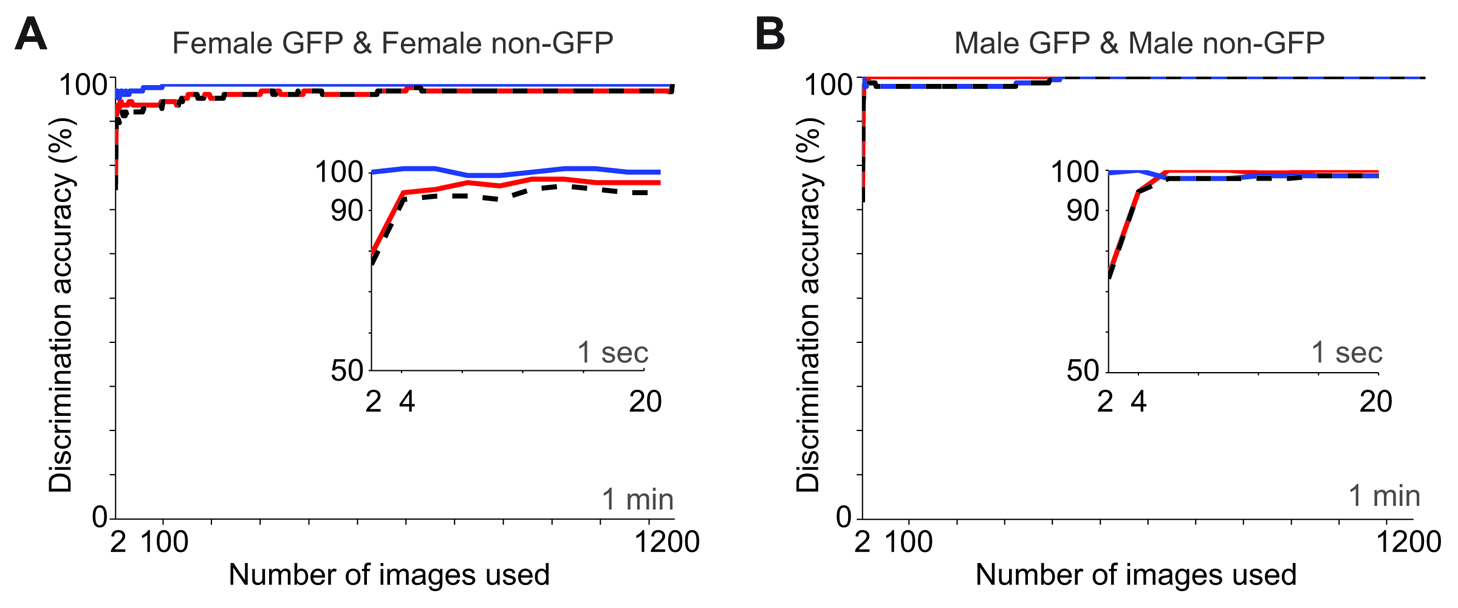

Supplement: Figure S6 — Discrimination accuracy as a function of the number of images used. Dependence of discrimination accuracy on the cumulative number of images used for data-averaging in A) female-female (n = 14 experiments; GFP females, n = 123; non-GFP females, n = 125) and B) male-male heterogeneous group experiments (n = 15 experiments; GFP males, n = 142; non-GFP males, n = 136). Metric weights are taken from homogeneous experiment analyses. Inset is a zoom into the first 20 images (note different y-axes). Red line: percent accurate non-GFP identifications, blue line: percent accurate GFP identifications, dashed black line: percent overall accuracy. (TIF) [file pone.0048381.s006.tif]

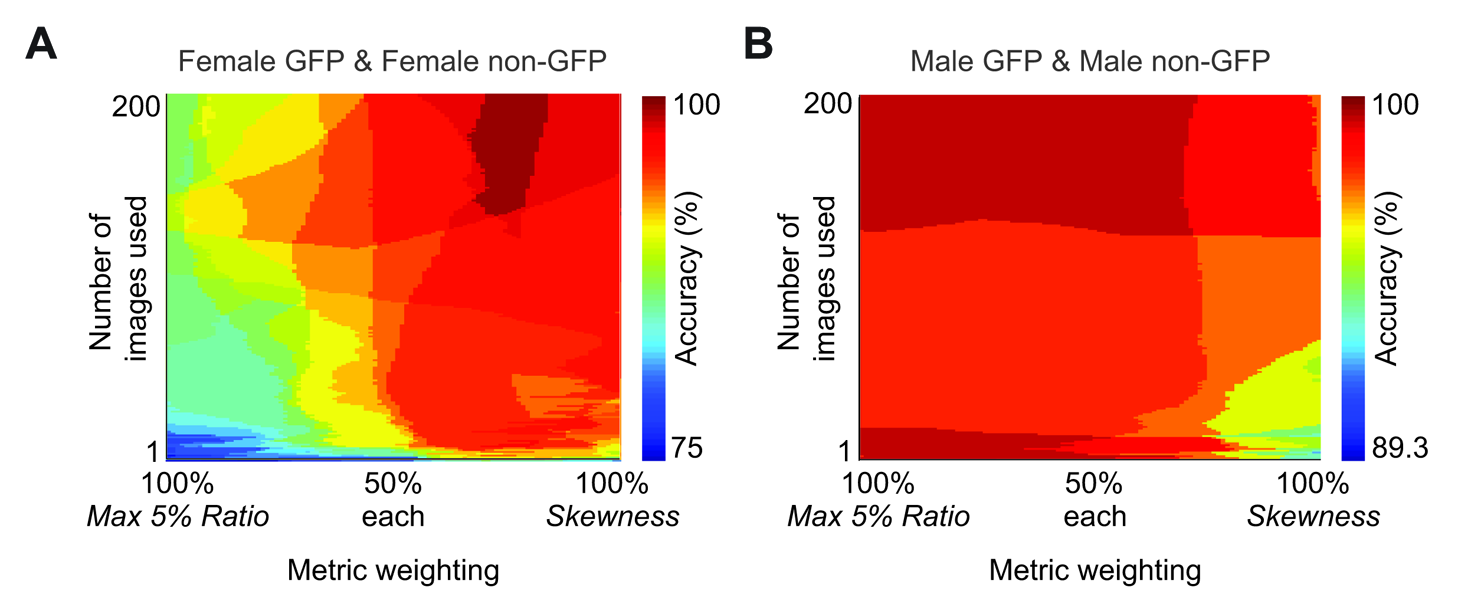

Supplement: Figure S7 — Discrimination accuracy when restricting FBI to the period after each experiment. Dependence of discrimination accuracy on metric weighting and the cumulative number of images used for data-averaging in A) female-female (n = 14 experiments; GFP females, n = 123; non-GFP females, n = 125) and B) male-male (n = 15 experiments; GFP males, n = 142; non-GFP males, n = 136) heterogeneous group experiments. Analyses employ only FBI data taken after each behavioral experiment. The number of flies expected in each genotype is incorporated into the discrimination algorithm. X-axes show the weighting of each metric. Y-axes show the cumulative number of images averaged for metric measurements. Color bars indicate the discrimination accuracy. (TIF) [file pone.0048381.s007.tif]
