# Supplementary material for: Fluorescence Behavioral Imaging (FBI) Tracks Identity in Heterogeneous Groups of Drosophila
Source: PLoS One. 2012 Nov 7;7(11):e48381. doi: 10.1371/journal.pone.0048381 (PMC3492344; doi:10.1371/journal.pone.0048381)
Supplement: Text S1 — Acquiring FBI data with sQuid and details regarding FBI Matlab m-files for post-processing FBI data. (PDF) [file pone.0048381.s008.pdf]

How to Use FBI Software

An accompaniment to the manuscript:

Fluorescence Behavioral Imaging (FBI) tracks identity in heterogeneous groups of Drosophila.
Pavan Ramdya, Thomas Schaffter, Dario Floreano, and Richard Benton

Acquiring FBI data with *sQuid*

sQuid is a general purpose C++/Qt software application permitting image acquisition from multiple cameras (in this case, an IR and a GFP camera) while controlling computer port output (in this case, parallel port control of LEDs). The software, installation guide, and user manual can be found at:

Download:
http://lis.epfl.ch/squid

For our experiments we run the cameras at 20 fps. To post-process data with the provided shell scripts, name the pin controlling the excitation LED "blue" in the *sQuid* player settings file.

Processing FBI data

STEP 1: sQuid raw data post-processing:
Following each experiment, it is necessary to build the required data types from raw *sQuid* imaging data. This can be accomplished by installing and using the provided shell scripts. Mplayer/mencoder must be installed first.

Download:
http://lis.epfl.ch/FBI

Script 1) batch_fluo_process
The user must run this script in the directory containing all of the experimental folders. This script runs the post-processing shell script *fluo_process* on each folder. This script is adapted from a script written for Ctrax batch processing (Kristen Branson).

Script 2) fluo_process
NOTE: The user must update the name of folders pointing to image files to the appropriate names associated with their camera ID numbers.

Makes a folder called "GFPimages" in the experiment directory.
Places all fluorescence images in this directory.
Makes a folder called "bkgd" in the experiment directory.

Places a subset of infrared (IR) images in this folder.
Makes a file "sequence.txt" containing all image names.
Converts IR .tif images into .jpeg for movie making
Makes an .avi video from IR images.
Deletes original image folders and images within.

STEP 2: Track movie.avi
Many different types of tracking software are available. For our experiments we used Ctrax [Branson et al. *Nat. Methods* 2009]. We tracked flies in the video file 'movie.avi'. This was followed by error fixing using the Matlab script 'fixerrors.m' provided by Ctrax developers. This yields a .mat file (e.g. movie_FIX.mat) with the structure, *trx*, containing fly specific structures with x, y, orientation, and size data needed for FBI processing. To accommodate another data format with the same information, some simple modifications must be made to FBI post-processing Matlab scripts.

STEP 3: Automatic genetic identity determination using Matlab m-files
The goal of FBI m-files is to use a tracked movie and corresponding GFP images to automatically determine the genetic identity of each tracked fly. These scripts have been developed with Matlab R2011b on Mac OSX 10.6.8.

Download:
http://lis.epfl.ch/FBI

Each experimental folder should have the following folders and files within:

1) A folder containing all raw GFP images:
experiment_directory/GFPimages/

2) GFP images within this folder with the following names:
*modelCamGFP*_HR_MIN_SEC_MSEC_blue.tif

3) A folder containing some IR images for background image creation:
experiment_directory/bkgd/

4) IR images within this folder with the following data names:
*modelCamIR*_HR_MIN_SEC_MSEC_pinID.tif

5) A matlab data file (.mat) containing tracking data (trx)
movie_FIX.mat
NOTE: This file naming convention can be different but must be noted in *assignParams.mat* for later processing

6) A text file (.txt) containing the sequence of image names
experiment_directory/sequence.txt

If all of these files are present, the user can run **FBI_Process_BATCH.m** to process one or more experiments in serial.

Post-processing FBI data

General Matlab scripts:

Script 1) /FBI_PreProcess/FBI_PrepTrx.m
Fixes first frame theta to second frame theta & calculates median fly sizes.
NOTE: default input is movie_FIX.mat
Input: *Folder directory with one experiment*
Data In: *.mat file with trx data*
Data Out: *trx_PREP.mat (*variable structure name: *trx)*

Script 2) /FBI_PreProcess/FBI_SequenceExtract.m
Extracts LED, timing, and frame name information from sequence.txt
NOTE: user must define camera name in line 83 for 'regexp'.
Input: *Folder directory with one experiment*
Data In: *sequence.txt file with image name data*
Data Out: *sequence.mat (*variables: *blueLEDoff; blueLEDon; ledSequence; nameSequence; timeSequence)*

Script 3) /FBI_PreProcess/FBI_AlignImages.m
Determines the scale, shift, & rotation values to align IR & GFP images. Takes user input to align.
NOTE: Can be performed once and used for multiple experiments by placing resulting *alignParams.mat* file in the experiment folder or in the directory containing all experiments for batch processing.
NOTE: assumes flipped GFP images vs. IR in the y-dimension: line 153.
NOTE: initialized vector is end-user specific. lines 175, 176
Input: *Folder directory with one experiment*
Data In: *images from /GFPimages/ and /bkgd/*
Data Out: *alignParams.mat* (var: *xAdd; yAdd; TFORM; height; width)*

Script 4) /FBI_PreProcess/FBI_MeasureFluo.m
Extracts image intensities at Front and Rear ROIs for each fly in GFP images.
Input: *Folder directory with one experiment*
Data In: *trx_PREP.mat; alignParams.mat; /GFPimages/image files*
Data Out: *trx_EXTRACT.mat* (var: *trx)*

Script 5) /FBI_Identify/FBI_SolveID.m
Calculates the likely identity of flies in experiment using metrics.
Input: *Folder directory with one experiment*

Data In: *trx_FBI.mat*
Data Out: *trx_ASSIGN.mat*

Matlab scripts for batch processing:
To process multiple experiments serially:

Script 1) /FBI_BatchProcess/FBI_Process_BATCH.m
Runs processing steps for each experimental folder in the parent directory.
NOTE: FBI_align_images.m can be performed for one experiment then *alignParams.mat* can be placed in Parent folder to use for all exp. directories.
NOTE: *assignParams.mat* should also be placed in the Parent folder in this manner.
Input: *Parent directory with all experiment directories*
Data In: *Parent directory name*
Data Out: *All outputs for each exp. directory.*

Matlab scripts for visualizing data:
Allow the user to visually inspect results:

Script 1) /FBI_Visualize/showtrx_GENO.m(fig)
Scroll through an experiment movie with a color-coded overlay of GFP and non-GFP flies. Red for non-GFP and blue for GFP.
NOTE: This is a modified version of Ctrax's showtrx.m. Therefore the Ctrax behavioral_microarray folder must be set in the Matlab path.
User Input: *location of .sbfmf & trx_ASSIGN.mat*
Data In: *.sbfmf; trx_ASSIGN.mat*

Script 2) /FBI_Visualize/FBI_movie_overlay_ROI_GFP_forward.m
Makes a movie in which ROIs are overlaid on GFP images from first frame onwards.
NOTE: user can define the length of the movie.
User Input: *Folder directory with one experiment*
Data In: *alignParams.mat; /GFPimages/image files/ ;trx_FBI.mat*
Data Out: *FBImovie_GFP_ROI.avi*

Script 3) /FBI_Visualize/FBI_movie_overlay_ROI_GFP_reverse.m
Makes a movie in which ROIs are overlaid on GFP images from last frame backwards.
NOTE: user can define the length of the movie.
User Input: *Folder directory with one experiment*
Data In: *alignParams.mat; /GFPimages/image files/ ;trx_FBI.mat*
Data Out: *FBImovie_GFP_ROI.avi*

Matlab scripts for fixing data manually:
Allow the user to change an assignment for a given fly.

Script 1) /FBI_Identity/FBI_set_identity.m
Allows the user to set the identities of all flies.
NOTE: Overwrites trx_ASSIGN.mat since assumes no solveID.
User Input: *Folder directory with one experiment*
Data In: *user defined trx.mat file*
Data Out: *trx_ASSIGN.mat*

Script 2) /FBI_Identity/FBI_fix_identity.m
Allows the user to fix a mis-identified fly.
NOTE: Opens and uses existing trx_ASSIGN.mat
User Input: *Folder directory with one experiment*
Data In: *trx_ASSIGN.mat*
Data Out: *trx_ASSIGN.mat*

Matlab parameter files:
Files to modify to set initial parameters:

File 1) /FBI_example_params/alignParams.mat
From **FBI_AlignImages.m**. Parameters to overlay IR and GFP images.

TFORM – data structure for scaling operation
Height – image height
Width – image width
xAdd – additional shift in x axis
yAdd – additional shift in y axis

File 2) /FBI_example_params/assignParams.mat
For **FBI_Process_Batch.m**. Parameters for processing and identifying.

numDRK - number of non-GFP flies expected
numGFP - number of GFP flies expected
ratio - ratio of metrics *max5% ratio* / *skewness*
trxName - tracking (Ctrax trx structure) data naming convention used
